# Early increase in blood supply (EIBS) is associated with tumor risk in the Azoxymethane model of colon cancer

**DOI:** 10.1186/s12885-018-4709-7

**Published:** 2018-08-13

**Authors:** Sarah Ruderman, Adam Eshein, Vesta Valuckaite, Urszula Dougherty, Anas Almoghrabi, Andrew Gomes, Ajaypal Singh, Baldeep Pabla, Hemant K. Roy, John Hart, Marc Bissonnette, Vani Konda, Vadim Backman

**Affiliations:** 1 Department of Biomedical Engineering, Northwestern University, Evanston, IL 60208 USA; 2 Center for Endoscopic Research and Therapeutics, University of Chicago Medicine, Chicago, IL 60637 USA; 3 Department of Gastroenterology, Rush University, Chicago, IL 60612 USA; 4 Department of Gastroenterology, Boston Medical Center, Boston, MA 02118 USA

**Keywords:** Colorectal cancer, Early increase in blood supply, Angiogenesis, Renin angiotensin system, Field effect of carcinogenesis

## Abstract

**Background:**

The present study aimed to investigate the role of blood supply in early tumorigenesis in colorectal cancer. We leveraged the renin angiotensin system (RAS) to alter colonic blood supply and determine the effect on tumor initiation and progression.

**Methods:**

To test the effect of blood supply on tumorigenesis, 53 male A/J mice were randomly assigned to one of three RAS modulation groups and one of two AOM treatments. The RAS modulation groups were I) water (RAS-unmodulated) as a control group, II) angiotensin-II and III) the angiotensin receptor blocker, Losartan. The mice in each group were then randomly split into either the saline control condition or the AOM-treated condition in which tumors were induced with a standard protocol of serial azoxymethane (AOM) injections. To monitor microvascular changes in the rectal mucosa during the study, we used confocal laser endomicroscopy (CLE) with FITC-Dextran for in-vivo imaging of vessels and polarization-gated spectroscopy (PGS) to quantify rectal hemoglobin concentration ([Hb]) and blood vessel radius (BVR).

**Results:**

At 12 weeks post-AOM injections and before tumor formation, CLE images revealed many traditional hallmarks of angiogenesis including vessel dilation, loss of co-planarity, irregularity, and vessel sprouting in the pericryptal capillaries of the rectal mucosa in AOM-Water tumor bearing mice. PGS measurements at the same time-point showed increased rectal [Hb] and decreased BVR. At later time points, CLE images showed pronounced angiogenic features including irregular networks throughout the colon. Notably, the AOM-Losartan mice had significantly lower tumor multiplicity and did not exhibit the same angiogenic features observed with CLE, or the increase in [Hb] or decrease in BVR measured with PGS. The AOM-AngII mice did not have any significant trends.

**Conclusion:**

In-vivo PGS measurements of rectal colonic blood supply as well as CLE imaging revealed angiogenic disruptions to the capillary network prior to tumor formation. Losartan demonstrated an effective way to mitigate the changes to blood supply during tumorigenesis and reduce tumor multiplicity. These effects can be used in future studies to understand the early vessel changes observed.

## Background

Colorectal cancer (CRC) remains the second leading worldwide cause of cancer-related deaths across both genders despite being highly treatable in its early stages. CRC is generally a slow process spanning decades from cancer initiation to diagnosis, and theoretically, provides considerable opportunity for early detection. Unfortunately, only 39% of patients are diagnosed at an early stage of the disease when the 5-year survival rate is 90%. The 5-year survival rate drops to 14% in the late stages of the disease when 21% of patients are diagnosed [1]. Given the important survival implications, there is an emerging interest in identifying tissue changes during the earliest stages of colonic carcinogenesis to improve diagnostic detection and risk stratification.

Primarily through neoangiogenesis, increased blood supply is a ubiquitous hallmark of cancer [2]. The phenomenon of *early increase in blood supply* (EIBS) in colon carcinogenesis has been demonstrated in both animal models and human trials [3–7]. In animal models, an increase in microvascular blood supply in premalignant stages in both the azoxymethane (AOM)-treated rat and the multiple intestinal neoplasia (MIN) mouse model of colonic tumorigenesis has been observed [3, 5–7]. Increases in hemoglobin concentration ([Hb]) and density of red blood cells were quantified with polarization-gated spectroscopy (PGS), a novel, depth-selective optical technique developed by our group [8]. Further studies using the AOM rat model demonstrated the role of nitric oxide synthase (iNOS) a potent angiogenic factor [4], as well as a shift in balance favoring angiogenic over anti-angiogenic factors in the premalignant stages [9]. Vasodilation and increased microvascular density (MVD), quantified through histological examination, were also detected as underlying causes of augmented blood content at the pre-adenoma stage [9]. These architectural and dynamic changes represent field carcinogenesis (also referred to as field effect) that could be exploited to improve diagnostic detection. Genetic and environmental factors that result in a localized malignant colonic transformation are known to induce more widespread biochemical and molecular changes throughout the colon [10]. Using PGS in vivo, we identified potential field carcinogenesis markers of blood supply and noted that in the microscopically normal rectal mucosa of patients harboring more proximal neoplasia, the superficial micro-circulation (within 100 μm of colonic luminal surface) was increased, even at distances greater than 30 cm from the malignant lesion [4]. Additional studies have confirmed markers of field carcinogenesis, including increased blood supply in microscopically normal-appearing rectal mucosa of patients with advanced adenomas in the more proximal colon [11–14].

While the importance of increased blood supply and the requirement for neoangiogenesis to support tumor growth are unequivocal [2, 15], the stage at which the process is initiated remains unclear. The classic “angiogenic switch” refers to the point at which tumor growth exceeds available blood supply such that hypoxia-induced changes induce angiogenic growth factors to promote neoangiogenesis [15]. Experimental models have yet to elucidate how changes in blood supply might precede hypoxic stimuli and directly shape subsequent tumorigenesis.

There are a multitude of pathways that regulate angiogenesis, including the renin-angiotensin system (RAS). Recent reviews have highlighted the emerging role of the RAS in regulating tumor growth and angiogenesis in experimental cancer models as revealed by angiotensin-converting-enzyme (ACE) inhibitors [16] and angiotensin receptor blockers (ARBs) [17]. The pro-angiogenic effects of angiotensin-II (AngII), including neovascularization [18] and arteriolization [19], are mediated at least in part by stimulating the production of growth factors, including vascular endothelial growth factor (VEGF). VEGF-A is up-regulated in most human cancers and is one of the most specific and potent angiogenesis factors known [20, 21]. AngII induces angiogenesis by activating AT1 subtype receptor (AT1R), but not the AT2 subtype. AngII-AT1 effects are mediated at least in part by the VEGF/eNOS-related pathway [22]. In a murine model of oxygen-induced retinal vascularization, AngII modulated VEGF-driven sprouting angiogenesis via AT1R [23]. Further demonstrating the role for AT1R in tumor angiogenesis, Chen et al. demonstrated that AngII promotes cell proliferation and upregulates VEGF-A expression in MCF-7 cells both in vitro and in vivo in a tumor xenograft murine model. They also reported a correlation between VEGF-A expression and increased microvascular density in human breast cancers [24]. Dougherty et al. showed that the RAS is up-regulated in a colitis model of colon cancer and that AngII stimulates colon cancer proliferation [25].

In order to gain a better understanding of the role of blood supply in shaping tumorigenesis, a method to modulate colonic blood supply independent of tumorigenesis is needed. In this regard, the RAS controls blood flow. The link between the AngII/AT1R signaling pathway and angiogenesis in recent studies, moreover, provide evidence that the RAS plays a role in colonic carcinogenesis. We exploited pharmacological manipulations of the RAS to investigate the role of blood supply during early stages of carcinogenesis. For these studies, colonic carcinogenesis was induced in mice with azoxymethane (AOM), a well-established model of chemical carcinogenesis and the RAS was modulated with either exogenous angiotensin-II or the angiotensin-II receptor (AT1) blocker, Losartan, to evaluate the effect of blood supply changes on tumorigenesis.

## Methods

### Animal model and study design

Animal experimental protocols were reviewed and approved by the Institutional Animal Care and Use Committee (IACUC) at University of Chicago (protocol number 72321). Mice were kept in IACUC approved and supervised housing in standard mouse plastic cages with bedding in social living settings with no more than five mice per cage. No wire caged floors were used. Mice were kept in a 12-h light/dark cycle. This study used 7–8 week old wild type A/J mice sourced from The Jackson Laboratory (USA) with susceptibility to the AOM carcinogen for tumor induction that we have previously employed in AOM treated rats to detect morphological alterations associated with field carcinogenesis [26, 27]. A total of 53 mice progressed in the study to be treated in one of three renin-angiotensin system (RAS modulation) groups: I) RAS-unmodulated control to serve as the baseline comparison for the other groups (18 mice); II) AngII injections (18 mice); and III) the ARB Losartan (17 mice). The angiotensin-II peptide was administered via injections (4 mg/kg of body weight i.p., 2× per week cyclically, with 2 weeks on and 2 weeks off) to increase the amount of circulating AngII. Losartan was administered in the drinking water (0.1 μg/kg/day) and provided ad libitum. It is an FDA approved drug in the class of angiotensin-receptor blockers (ARB) that competes with AngII for binding to the AT1 receptor and reduces the effect of AngII. The dosage was selected after reviewing literature and completing a short-term pilot study to evaluate the efficacy of drugs for each group (data not shown).

The mice in each group were randomly assigned to either the saline control condition (*n* = 6 mice RAS-unmodulated, 10 mice given AngII and 7 mice given Losartan) or the AOM-treated condition (*n* = 12 mice RAS-unmodulated, 8 mice given AngII and 10 mice given Losartan). Tumors were induced with a standard protocol of serial AOM injections (7.5 mg/kg of body weight i.p.) administered weekly for 6 weeks. RAS modulation (unmodulated, AngII injections, or Losartan) was started 2 weeks prior to the first AOM injection and continued throughout the course of the experiment. All 53 mice were included in all analysis and reported results.

We examined microvascular changes using a combination of techniques and mice were sedated with xylazine and ketamine (i.p.) for all procedures. PGS and confocal laser endomicroscopy (CLE) measurements were taken at the beginning of the study (0 weeks), immediately prior to AOM injections (2 weeks), and then continued every 4 weeks post-AOM injections (12 weeks until 24 weeks). Colonoscopy was performed at the last 2 time-points (20 weeks and 24 weeks), when we expected to detect tumors based on previous studies.

### Gross assessment by colonoscopy

At indicated time-points (20 and 24 weeks), a small animal rigid colonoscope (Karl Storz, Germany) was inserted per rectum under sedation and air injected via a syringe to insufflate the colon. The scope allowed visual examination of the mouse colon and was advanced under direct visualization at least 3 cm proximal to the anus. Still images were captured on withdrawal at 3, 2, 1, and 0.5 cm from the anal verge. Careful inspection for tumors or visible lesions was made on withdrawal. All suspicious lesions were photographed and noted with an estimate of tumor size and distance from the anal verge and clock position, with 12 o’clock marking the tail. Mice were categorized into “Early tumor formers” and “Late tumor formers” based on the time-point when lesions were detected on colonoscopy. “Early tumor formers” were mice with lesions detected at the 20-week colonoscopy examination. “Late tumor formers” were mice with lesions detected at the final colonoscopy examination, at 24 weeks or at the time of sacrifice since they developed lesions at a later time-point.

### Microvascular blood supply assessment by PGS

Polarized-gated spectroscopy (PGS) has been described in previous publications [4, 8, 11, 28]. The PGS fiber-optic probe enables quantification of the blood supply within the pericryptal capillaries of the colonic mucosa (100 to 200 μm mean penetration depth). Hemoglobin (Hb) content was calculated from the PGS signal using an algorithm described previously [4, 8, 29]. This analysis quantified oxygenated and deoxygenated Hb concentrations, as well as average blood vessel radius (BVR) as indirect measures of microcirculation. For measurement acquisition, the PGS probe was inserted per rectum and placed in gentle contact with the normal-appearing mucosal surface for 25 random measurements within the rectum. Each measurement was acquired from a unique tissue site within the rectum. In mice with rectal tumors, measurements were acquired in rectal mucosa free of tumors. These data were analyzed separately from mice with a normal appearing rectum that harbored neoplasia in more proximal areas of the colon. The latter were used to assess generalized changes in microvasculature related to the field effect.

### Microvascular structure assessment by CLE

Dynamic microscopic imaging was performed with a confocal laser endomicroscopy (CLE) fiber-optic probe system developed by Mauna Kea Technologies (MKT, Cellvizio, Paris, France). Briefly, the probe is placed in contact with the mucosa and combined with administration of a contrast agent, delivers high quality real-time video images of the colonic mucosal microvessels [30]. The video rate is 12 images/sec and the lateral resolution is 1 μm with an optical slice of 10 μm. In order to visualize vessels, mice were administered 100 μl FITC-D (70, 000 MW) as contrast agent via tail vein injection prior to inserting the CLE probe per rectum to capture video sequences at 3, 2, 1, and 0.5 cm from the anal verge. Representative static images were selected from the recorded video sequences at each time-point in each group and confirmed tumors and normal appearing mucosa were also examined histologically. Three investigators, blinded to treatment groups and conditions, performed offline analysis to score images using the 10-point system we have previously described [31], based on loss of co-planarity, vessel dilation, sprouting, irregular vessel pattern and extravasation. The final image score was an average of the scores of the three investigators.

### Dil imaging

Blood vessels were directly labeled by using an aqueous solution containing 1,1′-dioctadecyl-3,3,3′,3′-tetramethylindocarbocyanine perchlorate (DiI) (D-282, Invitrogen/Molecular Probes; 42,364, Sigma-Aldrich) [32]. Sedated mice were sacrificed by CO_2_ asphyxiation, followed by cervical dislocation. The abdominal cavity was opened via a transverse incision and the distal abdominal aorta was exposed. The proximal aorta was clamped in order to block the flow to the upper body. The Dil solution was injected into the distal aorta using the perfusion device (consisting of two three-way stopcocks, a 30-gauge butterfly needle and three 10-ml syringes). The perfusion order included: 1) 5 ml of PBS at the rate of 1–2 ml/min; 2) 5–10 ml of the DiI solution at the rate of 1–2 ml/min; and 3) 5–10 ml of the fixative at the rate of 1–2 ml/min. After perfusion, colon tissue was harvested and kept in 4% paraformaldehyde for 48 h. Stained and fixed, vessels were visualized with a Leica confocal microscope.

### Tissue acquisition and assays

At 24–28 weeks after carcinogen administration, animals were sacrificed. The entire colon (cecum to rectum) was excised and opened flat. Tumors were enumerated and sized and location was recorded in situ and lesions excised via punch biopsy for histological analysis. A section of the distal colon tissue was harvested and subjected to real-time PCR and Western blotting analysis for VEGF-A expression.

### RT-PCR

RNA was extracted and *VEGF-A* mRNA was quantified by real time PCR. RNA was extracted from snap frozen tissue using Qiagen miRNeasy Mini Kit that captures total RNA including miRNA. Samples were homogenized with a Polytron and loaded onto an RNA-binding spin column, washed, digested with DNase I and collected in 30 μl elution buffer. RNA samples were examined by Agilent chip for RNA purity and quantified by Ribogreen. RNA (100 ng) was reverse transcribed into cDNA using high capacity reverse transcription kit in 20 μL total volume. Incubation conditions were 25 °C for 10 min, 37 °C for 120 min, and 85 °C for 5 min. The resulting first-strand complementary DNA (cDNA) was used as template for quantitative PCR in triplicate using fast SYBR green master mix kit. Oligonucleotide PCR primer pairs were designed from published mouse sequences in the GenBank database using Primer3 [33]. The primer sequences are for forward VEGF-A F1: 5’-AAF GAG GAG GGC AGA ATCAT-3′ and reverse VEGF-A R1: 5’-TCC AGG CCC TCG TCA TTG-3′. Reverse transcribed cDNA (1:10 dilution) and primers were mixed with fast SYBR green master mixture in 20 μl. Reactants were initially heated to 95 °C for 20 s followed by 40 cycles as follows: denaturation step at 95 °C for 10 s, annealing step at 55 °C for 15 s and extension step at 60 °C for 30 s. PCR amplification was verified by melting curve analysis and predicted electrophoretic mobility of the PCR amplicon on confirmed 3% agarose gel. There were no detectable amplifications in the negative control samples (reactions lacking reverse transcriptase or reactions without DNA template). The data were analyzed using the comparative 2exp(-ΔΔCt) method, and mRNA abundance normalized to β-actin mRNA and expressed as fold-control.

### Western blot analysis

Proteins extracted in Laemmli buffer were assayed for VEGF-A by Western blotting. Freshly harvested colons were placed in 2X sodium dodecyl sulfate–containing Laemmli buffer and sonicated for 1 min with a Branson microprobe (Branson, Danbury, CT). After sonication, the samples were boiled for an additional 5 min and centrifuged to remove insoluble material. Protein concentrations were determined using RC-DC Protein Assay (BioRad Laboratories, # 500–90,119, Hercules, CA). Proteins were separated by glycine-based SDS-PAGE system (12% gel) and transblotted onto an Immobilon-P membrane (Millipore, Bedford, MA) at 75 mA overnight using a minigel transfer apparatus (Hoefer, Holliston, MA). Membranes were stained with 0.05% India ink to assess comparable protein loading and transfer. The membranes were blocked with Tris-buffered saline containing 0.05% Tween-20 and 5% dry milk and incubated overnight with primary antibody (Santa Cruz Biotechnology SC-7269 Monoclonal VEGF A, Dallas, TX) in 5% dry milk (pH 7.5) at a concentration of 1:150 overnight followed by incubation in secondary antibody (GE Healthcare ECL antimouse IgG NA931V, Marlborough, MA) at a concentration of 1:300 for 1 h at room temperature. Blots were re-probed for β- actin to assess loading (Sigma-Aldrich, St. Louis, MO). Densitometry was performed using the UN-Scan-it gel software package V5.3. (Silk Scientific, Inc., Orem, UT).

### Histology & Immunohistochemistry (IHC)

Freshly harvested colons were fixed overnight in 4% formaldehyde in PBS (pH 7.2), then processed and embedded in paraffin wax. 5 μm sections were mounted on Vectabond-coated Superfrost Plus slides and stained with hematoxylin and eosin. An expert gastrointestinal pathologist reviewed all histology and identified gross and microscopic foci of tumors. VEGF was analyzed by IHC to assess cell of origin, including colonic epithelial cells, myofibroblasts and endothelial cells. For VEGF-A staining sections were heated to 60 °C for 1 h, deparaffinized by three washes for 5 min each in xylene, hydrated in a graded series of ethanol washes and rinsed in distilled water. Epitope retrieval was performed by steaming for 15 min in 0.01 M citrate buffer (pH 6), followed by three washes for 2 min each in Tris-buffered saline with 0.1% Tween-20 (TBST). Endogenous peroxidase activity was quenched with methanol/H_2_O_2_ solution (0.5%). Sections were washed three times in TBST for 2 min each and blocked in protein block for 20 min. Sections were incubated with primary antibody (1:50 dilution of anti-VEGF A antibody (Santa Cruz Biotechnology SC-152, Dallas, TX) for 1 h at room temperature. After three washes in TBST, slides were incubated at room temperature with 1:200 dilution of biotinylated secondary antibodies for 30 min. Antigen–antibody complexes were detected using 3,3′-diaminobenzidine as substrate and horseradish peroxidase–labeled DAKO EnVision™ + system. After washing in distilled water, slides were counterstained with Gill’s III hematoxylin, rinsed with water, dehydrated in ethanol and cleared with xylene.

### Statistical analysis

For non-parametric analysis, the Mann-Whitney test was performed. For comparison of normally distributed continuous variables, the Student’s t-test was performed. Differences with *p* < 0.05 were considered statistically significant. All statistically significant relationships in each dataset are noted in figure captions as well as discussed in the text.

## Results

### Tumorigenesis

**Fig. 1:**
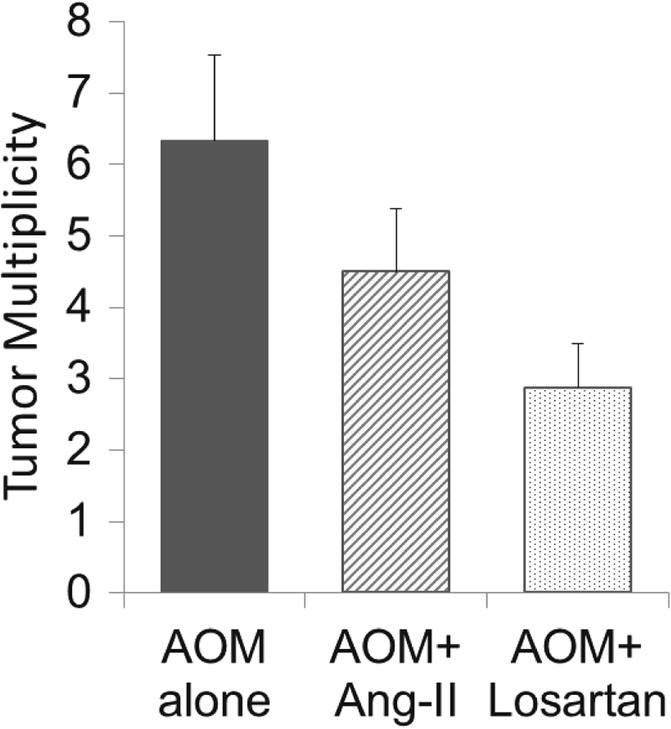
Tumor multiplicity for each of the AOM-treated groups. The AOM alone (no RAS modulation) group had the largest multiplicity (6.3), followed by AOM + AngII group (4.5), and AOM + Losartan group with the lowest (2.9). The AOM + Losartan group was significantly lower than AOM alone (*p* = 0.018)

A total of 166 elevated lesions (from *n* = 30 mice) were identified and submitted for histology as presumptive tumors. Tumors were confirmed microscopically and in areas of extensive transformation efforts were made to distinguish single large lesions from colliding lesions. A total of 116 tumors were identified that comprised 72 adenomas and 44 carcinomas. Total tumor incidence was 9/12, 8/8 and 8/10 for AOM alone, AOM + AngII and AOM + Losartan, respectively. As shown in Fig. 1, tumor multiplicity trended such that mice receiving AOM + Losartan (2.9) had significantly lower multiplicity compared to the AOM alone group (6.3) with a *p* value of 0.018. The AOM + AngII group was intermediate (4.5) with no statistically significant difference compared to the AOM alone group (*p* = 0.28).

### Effects of RAS modulation and AOM treatment on VEGF-A protein

**Fig. 2:**
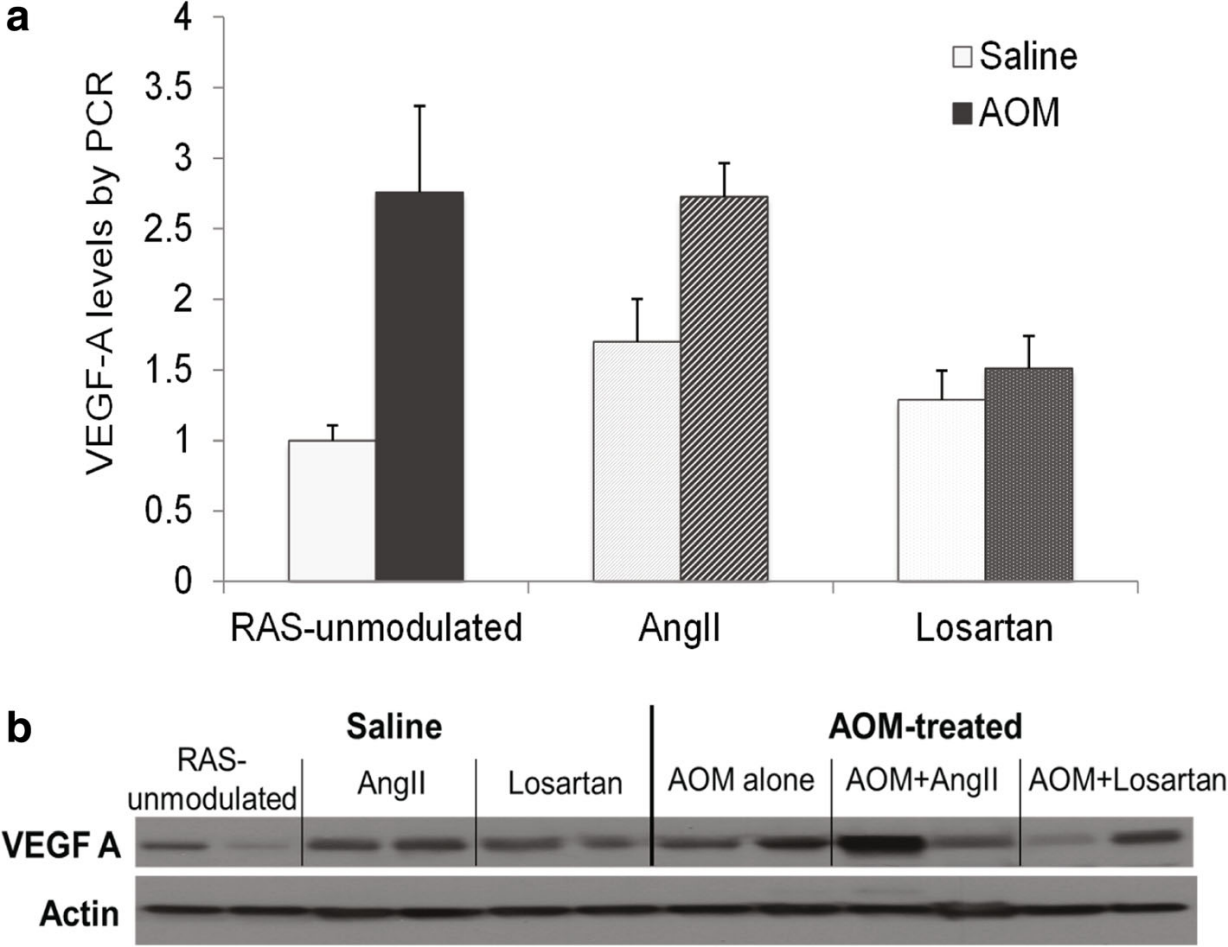
**a** VEGF-A mRNA expression as assessed by RT-qPCR with data normalized to saline controls in the RAS-unmodulated group. Overall, RAS modulation and AOM-treatment up-regulated VEGF in colonic mucosa (ANOVA *p* = 0.004). AOM-treatment alone upregulated VEGF-A 2.8-fold in AOM alone group (*p* = 0.03), 2.7-fold in AOM + AngII group (*p* < 0.001), and 1.5-fold in AOM + Losartan group (*p* = 0.08). Although not significant, there was a trend for Losartan suppressing VEGF-A expression in AOM-treated mice. Error bars represent standard error of the mean. **b** Representative Immunoblot of VEGF-A protein levels for each group. Overall, RAS modulation increased VEGF-A protein levels compared to saline treated mice in the RAS-unmodulated group. AOM-treatment also increased VEGF-A protein levels among the RAS-unmodulated and AOM + AngII groups. The AOM + Losartan group exhibited a decrease in VEGF-A compared to AOM alone or AOM + AngII

*VEGF-A* mRNA levels were quantified by real time PCR and all groups were normalized to the saline treated (AOM vehicle) controls that comprised the RAS-unmodulated group (mice receiving no AngII or losartan). Both RAS modulation and AOM-treatment up-regulated VEGF-A in the colonic mucosa (Fig. 2a, ANOVA *p* = 0.004). Within the saline treated mice (no AOM), VEGF-A was up-regulated 1.7-fold in the AngII group, and 1.3-fold in the Losartan group compared to the RAS-unmodulated group. AOM treatment up-regulated VEGF-A in all of the RAS modulation groups: 2.8-fold in the AOM alone group compared to 2.7-fold in the AOM + AngII group, and 1.5-fold in the AOM + Losartan group (Fig. 2a). This generalized increased VEGF-A reflects a field effect in the colonic mucosa. There was a trend, although not significant, for Losartan to down-regulate VEGF-A among the AOM-treated mice (2.8 vs. 1.5).

In RAS-modulated mice without AOM, colonic mucosal VEGF protein levels were greater than the RAS-unmodulated group without AOM as shown in a representative blot in Fig. 2b. Compared to RAS unmodulated mice without AOM, VEGF protein levels were increased in the AOM alone and AOM + AngII groups. Interestingly, in the group given AOM + Losartan, there was a decrease in VEGF protein levels compared to the AOM alone treated group.

### Ex vivo vessel architecture as assessed by Dil staining

**Fig. 3:**
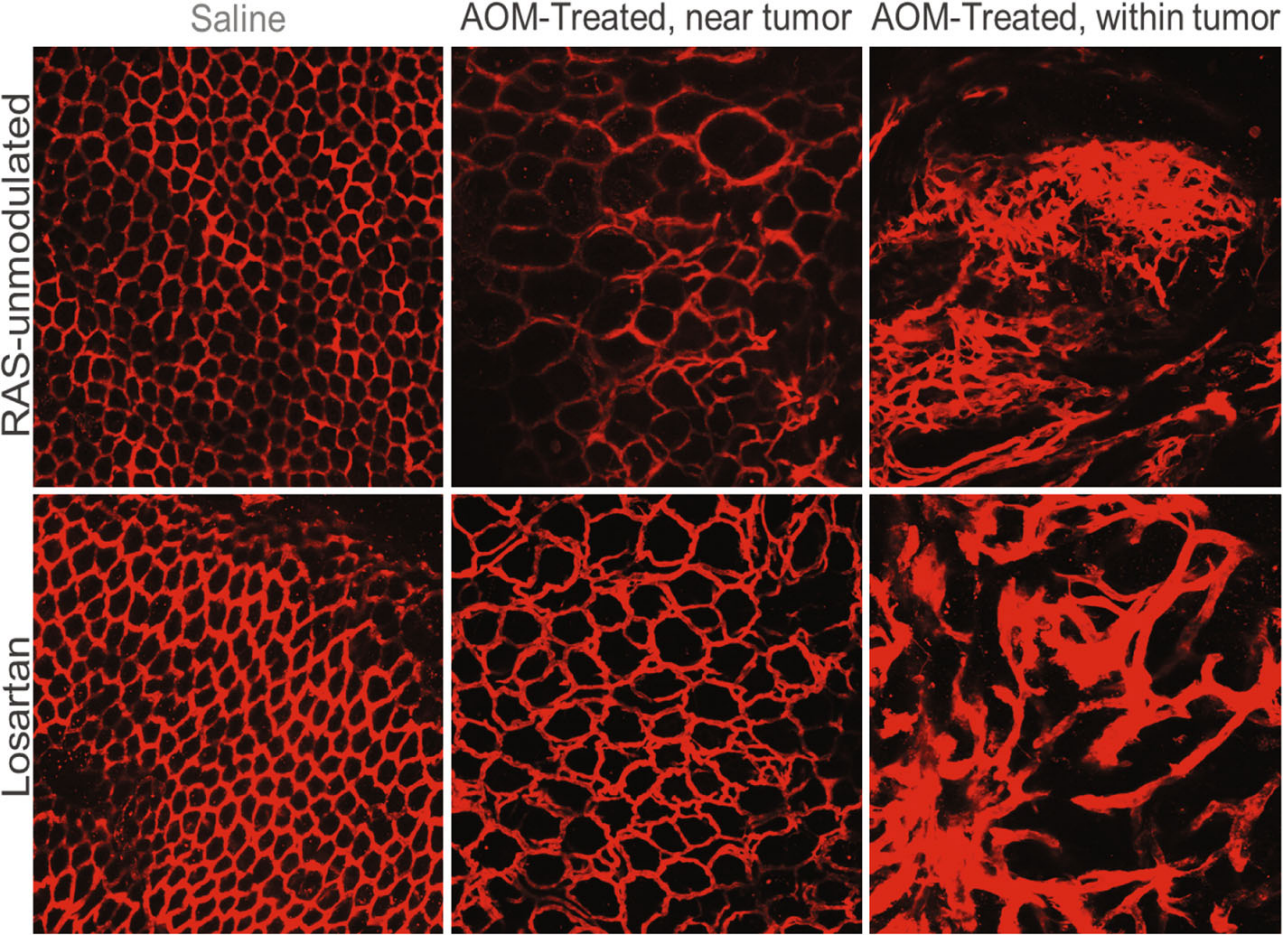
Ex vivo microvessel images with DiL staining. The saline-treated animals (AOM controls) in both RAS-unmodulated and Losartan groups (left column) exhibit the expected uniform, honeycomb structure. Adjacent to the tumor (middle column), there are minor disruptions to the vessel network, including tortuous and sprouting vessels. The vascular network within tumors (right column) is completely disrupted with vessels of varying size and a chaotic structure

Representative images from the subset of mice randomly selected for ex vivo blood vessel imaging with Dil staining are shown in Fig. 3. As expected, the colon microvasculature from the saline animals (Fig. 3, left column) exhibited the uniform, honeycomb pattern observed with in vivo CLE images (Fig. 6, left column) and reported in literature [34]. The AOM-treated mice exhibited abnormal vascular networks, both within tumors and adjacent to tumors in normal-appearing mucosa. In areas adjacent to tumors (Fig. 3, middle column), vessels maintained some aspects of the honeycomb structure, but varied in size, tortuosity and sprouting features generating irregular vascular patterns. The vascular network within tumors (Fig. 3, right column) was completely disrupted, comprised of irregular vessels of varying size and chaotic arrangements deviating from the normal co-planar organization. These aberrant vessels possess features described previously in tumor neoangiogenesis [15].

### PGS detects EIBS associated with tumor development

**Fig. 4:**
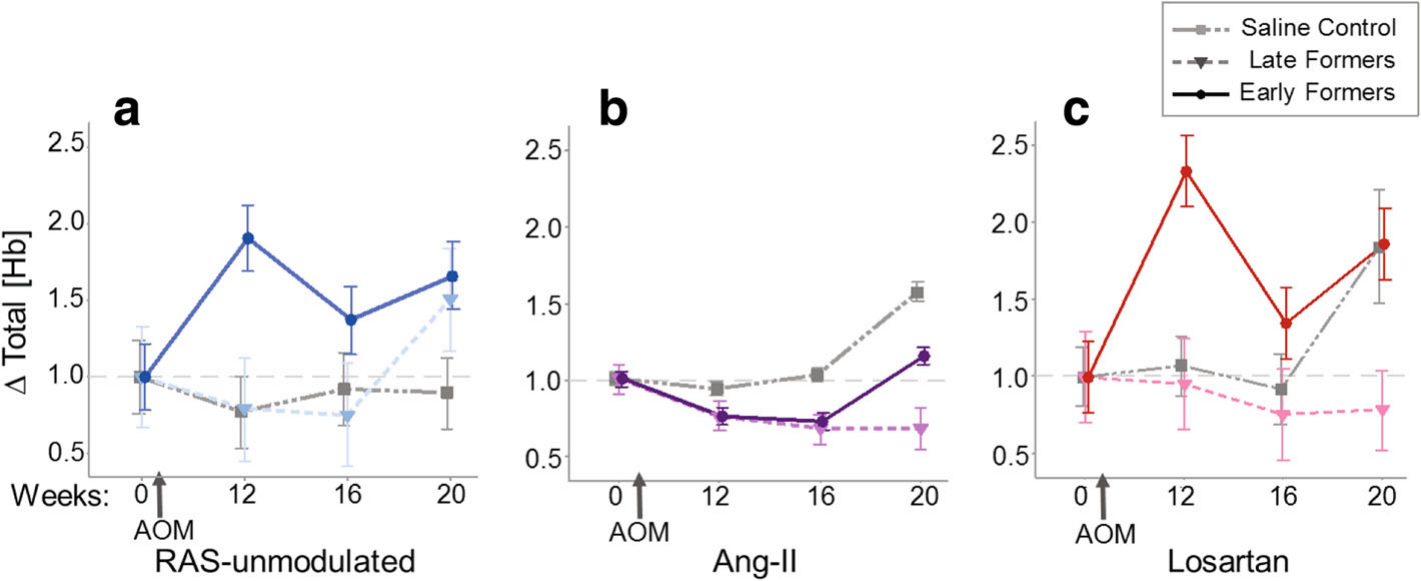
Rectal microvascular perfusion measured by PGS at indicated times during tumorigenesis. Total [Hb] was individually normalized per mouse by data recorded at week 0 for the (**a**) RAS-unmodulated; (**b**) AngII; and (**c**) Losartan groups. Mice were categorized based on colonoscopy reports of suspected lesions at 20 wks (Early tumor formers), 24 wks (Late tumor formers). The Early tumor formers in the (**a**) AOM alone group and (**c**) AOM + Losartan group exhibit an early increase in blood supply at wk. 12, a time-point preceding any visible tumors. Error bars represent standard error of the mean

Microvascular perfusion in the rectal mucosa was measured by PGS at multiple indicated time-points throughout the course of the study. At the beginning of the study (week 0) all mice exhibited similar total Hb concentrations ([Hb]) (ANOVA *p* = 0.98). Changes in total [Hb] were normalized individually for each mouse based on the total [Hb] recorded at wk. 0. Changes in [Hb] over time are shown in Fig. 4. The RAS-unmodulated control mice (no AOM) maintained a consistent rectal [Hb] throughout the course of the study (ANOVA *p* = 0.35). In order to assess blood supply as an indicator of tumor development, AOM-treated mice were categorized based on colonoscopy reports. Colonoscopy was performed on each mouse at 2 time-points after study initiation: 20 wks and 24 wks (time of sacrifice) and suspicious lesions were recorded. Based on the earliest point when a lesion was noted, mice were divided into 2 categories: 1) early tumor formers, with suspected lesions prior to 20 wks; or 2) late tumor formers, with lesions noted after 20 wks.

For the AOM alone group (Fig. 4a), mice in the “Early tumor formers” subcategory exhibit an increase in blood supply as early as 10 weeks post-AOM injections (*p* = 0.04) and these levels remain elevated throughout the course of the study. The mice in the “Late tumor formers” subcategory exhibited an increase in blood supply at a later time-point. The most important observation is that in both cases, the increase in blood supply is observed prior to the formation of any lesion. This data is consistent with our previous studies involving the AOM-model [3, 9, 26] that demonstrated an early increase in blood supply (EIBS) associated with risk of neoplasia.

In the AngII group (Fig. 4b), the saline-treated mice (AOM controls) blood supply was elevated above RAS-unmodulated mice (Fig. 4a) indicating that the exogenous AngII alone could potentially increase the rectal blood supply. The AOM + AngII mice did not display any significant trends in blood supply associated with tumor development.

In the AOM + Losartan group (Fig. 4c), mice that developed early lesions (prior to 20 wks) also demonstrated an early increase in blood supply similar to the AOM alone group with early tumors. In contrast an early increase in blood supply was not observed in the late tumor formers in this group.

### PGS detects decrease in vessel radius associated with tumorigenesis

**Fig. 5:**
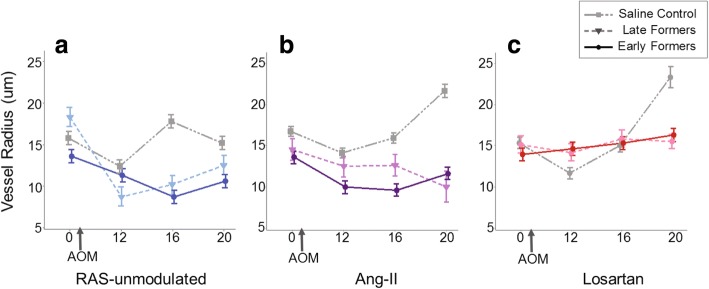
Average blood vessel radius (μm) measured by PGS. **a** Vessel radii in the saline (RAS-unmodulated, AOM control) group remain relatively constant over the study; whereas the AOM-treated mice develop a decrease in vessel radius, most pronounced at the 16 wks time-point. **b** The AOM + AngII mice also show a decrease throughout the study. Decreasing vessel size may reflect sprouting new vessels. **c** The AOM + Losartan mice maintain a relatively constant vessel radius over the study duration. Error bars represent standard error of the mean

**Fig. 6:**
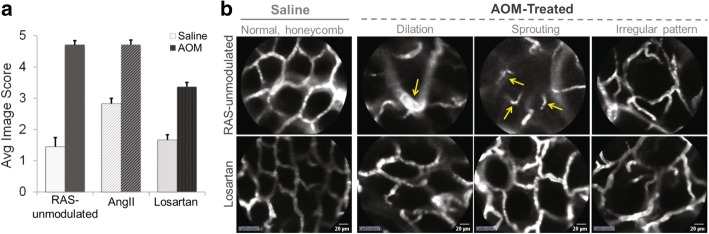
**a** Average CLE image scores at 24 weeks. Representative CLE images were evaluated with our 10-point system based on loss of co-planarity, vessel dilation, sprouting, irregular vessel pattern and extravasation. Images of the RAS-unmodulated group displayed the expected microvessel patterns. The higher scores of images from the AOM-treated mice indicate microvessel abnormalities in the RAS-unmodulated and AngII groups (*p* = 0.02 and p = 0.01, respectively). Losartan treatment mitigated vessel derangements observed with no significant difference between the Losartan alone (saline) and AOM + Losartan groups (*p* = 0.49). Error bars represent standard error of the mean. **b** Representative CLE images at 16 weeks. The left-most column (column 1) shows representative images from saline-treated mice in both RAS-unmodulated (upper panels) and Losartan (lower panels) groups. The right columns (columns 2–4) show images from AOM-treated mice in RAS-unmodulated (upper row) and Losartan (lower row) groups prior to tumor development. At 16 weeks, the RAS-unmodulated group (upper row) displays vessel dilation, the sprouting of new, tiny vessels and an overall disruption in the normal, honeycomb pattern observed in the images of saline mice (i.e. left column). In the Losartan group (lower row), while there are a few aberrations in vessel patterns, the dilation and sprouting changes are not prominent

Another parameter assessed by PGS is the average vessel radius calculated from the spectra of the optically interrogated tissue. Fig. 5 shows the average blood vessel radius measured by PGS for each of the 6 treatment groups (saline controls and AOM). For the RAS-unmodulated group, as in the case of total [Hb], the saline controls do not show any significant blood vessel radius change over time (ANOVA *p* = 0.35). However, the AOM alone mice reveal a decrease in vessel radius, most significant at 16 wks post-AOM injections (*p* = 0.01), which then, slowly increased to match saline-treated (AOM controls) in the RAS-unmodulated mice by the end of the study (Fig. 5a). This correlates with the small vessels observed in the CLE images (shown in Fig. 6) and potentially indicates the sprouting of new vessels, or neoangiogenesis. In the AngII group, the AOM + AngII mice also display a decreasing trend in vessel radius throughout the study (Fig. 5b). These vessel size changes could reflect vasoconstriction due to the exogenous angiotensin-II and/or new vessel sprouting by VEGF induction. In the Losartan group, there are no significant changes over time among the Losartan alone or AOM + Losartan mice (Fig. 5c). In the case of the AOM + Losartan mice, this could reflect the potential anti-angiogenic properties of the ARB blocking the effects of the carcinogen.

### In-vivo microvessel architecture assessed longitudinally with CLE

CLE video images revealed abnormalities in the pericryptal capillaries throughout tumorigenesis. At week 0, all CLE microvessel imaging depicted a uniform, honeycomb pattern expected in normal colon (similar to images shown in left column of Fig. 6b). As early as week 12 of the study (corresponding to 10 wks post-AOM injection) prior to any detectable tumors, vessel anomalies were observed in the AOM-treated mice compared to RAS-unmodulated control group, including the presence of tiny vessels (sprouting) and irregular or distorted patterns in the honeycomb structure. Fig. 6b illustrates some of the early alterations in the microvascular network at week 16 (prior to tumor formation) in mice from RAS-unmodulated (top row) and Losartan (bottom row) groups. In the RAS-unmodulated group, the microvessel abnormalities were more prevalent with dilation of vessels, increased sprouting and disruptions in the vascular network. The Losartan group maintained a more regular vessel network with little sprouting noted, potentially indicating the anti-angiogenic properties of the ARB. At later time-points, the CLE imaging within and adjacent to tumors revealed microvascular alterations consistent with our previous studies on tumor angiogenesis (data not shown) [31]. Fig. 6a represents the average CLE image scores for each group calculated using the semi-quantitative 10-point scoring system we developed to assess microvessel features including loss of co-planarity, vessel dilation, sprouting, irregular vessel pattern and extravasation [31]. Among saline-treated mice, the AngII group (*P* = 0.05), but not the Losartan group (*p* = 0.2) exhibited more alterations in the microvascular network compared to the RAS-unmodulated group. Furthermore, there was a significant difference between the saline-treated and AOM-treated mice in both the RAS-unmodulated and AngII groups (*p* = 0.02 and *p* = 0.01, respectively), but not in the Losartan group (*p* = 0.49). This indicates that Losartan treatment mitigated the vessel derangements observed in the other 2 groups.

### Baseline blood supply levels prior to AOM treatment may influence tumorigenesis

**Fig. 7:**
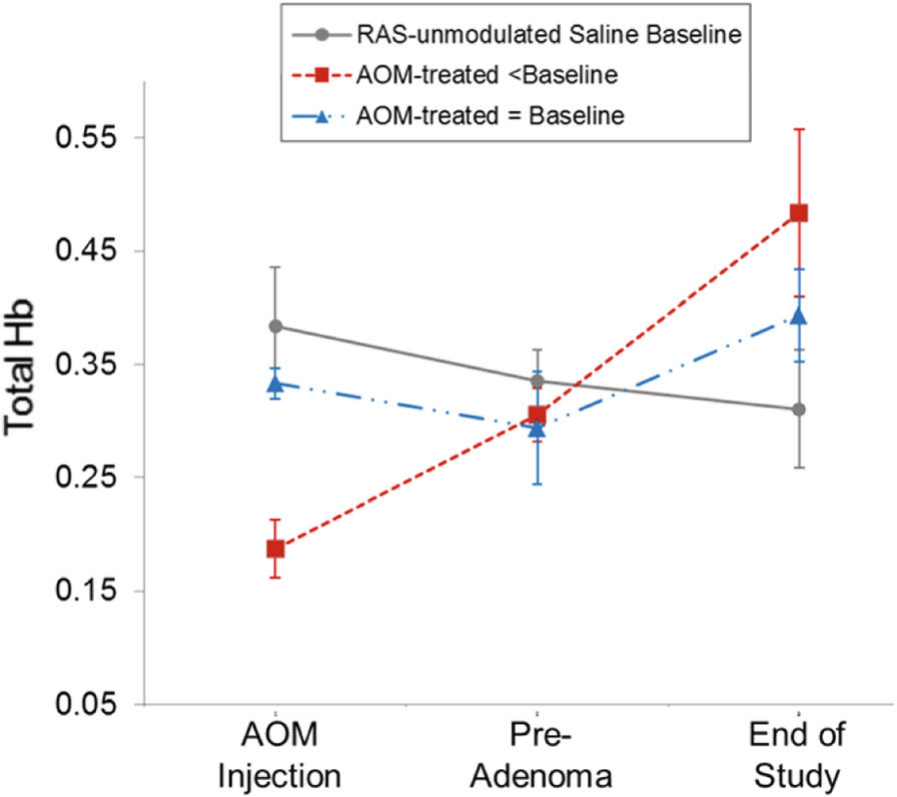
Time dependent changes in rectal [Hb] as assessed by PGS in mice grouped by blood supply level prior to tumor induction. Subset of AOM-treated mice (*n* = 16) were separated based on their absolute [Hb] compared to saline, RAS-unmodulated group (baseline) measured at wk. 2. AOM-treated mice within 30% of the mean rectal [Hb] in baseline group were considered similar to the baseline (blue line). Those with [Hb] < 70% baseline were grouped together as “Lower” blood supply (red line). Mice with lower [Hb] exhibited EIBS associated with tumor formation. AOM-treated mice with similar [Hb] to baseline did not show an increase in rectal [Hb] associated with tumors. Error bars represent standard error of the mean

The absence of an increase in blood supply associated with tumors from the groups with RAS modulation led us to examine data based on the blood supply level recorded immediately prior to AOM injections. A subset of mice (*n* = 22) had PGS measurements recorded at the week 2 time-point (data not shown on previous figures). Of these mice, 6 belong to the saline-treated, RAS-unmodulated group and 16 belong to AOM-treated groups. For this analysis, the mean of RAS-unmodulated, saline controls at week 2 served as the “baseline group” and RAS modulation was ignored when dichotomizing the AOM-treated mice. AOM-treated mice within 30% of the mean rectal [Hb] of saline controls (*n* = 8) were labeled “similar” to the baseline. Those with [Hb] < 70% baseline group were labeled “Lower” blood supply (*n* = 5). The absolute rectal [Hb] at 3 distinct points over the course of the study are shown for each category in Fig. 7. The points are (1) “AOM-injection” corresponding to measurements recorded immediately prior to treatment with the AOM carcinogen (wk 2); (2) “pre-adenoma” corresponding to a time-point prior to any lesions noted on colonoscopy (wk 16); and (3) “end of study” corresponding to the last measurement point for each animal. AOM-treated mice with lower [Hb] (Fig. 7, red line) exhibited an early increase in blood supply associated with tumor development, regardless of RAS manipulation. Interestingly, AOM-treated mice with a similar [Hb] to “baseline” (Fig. 7, blue line) tracked closely with “baseline” group throughout the study, regardless of tumors. It should be emphasized that only this small subset of mice (*n* = 16) from the total number of AOM-treated mice that survived to the final time-point (*n* = 30) had data recorded at week 2. These preliminary results, while provocative, should be interpreted with caution.

## Discussion

In this study, we used the AOM model that mimics many of the molecular and cellular changes observed in human colorectal cancer to evaluate changes during tumor initiation and progression. We have shown an early increase in rectal blood supply of AOM-treated mice that developed colon tumors. We observed changes within the microvascular structure that occur prior to tumor development. We have also demonstrated that modulating the RAS system with the ARB inhibitor, Losartan suppressed the changes in blood supply and decreased tumor multiplicity. A novel aspect of this study was our application of two independent technologies to assess angiogenesis throughout the course of the study using minimally invasive methods. PGS quantified rectal hemoglobin concentration ([Hb]) and average blood vessel radius (BVR) within the superficial mucosa as a measure of blood supply to the colon. CLE provided in-vivo dynamic imaging of the pericryptal microvasculature to assess derangements in vessel networks. Together these results agree with our previous reports of an early increase in blood supply (EIBS) associated with colonic neoplasia and support our hypothesis that changes in blood supply play an important causal role during the early stages of carcinogenesis.

As noted earlier, field effects refer to widespread cellular and molecular changes in the colon resulting from genetic and environmental alterations that contribute to malignant transformation [10]. Our previous studies have established PGS as a sensitive tool to detect field changes in rectal blood supply associated with concomitant colonic neoplasia [4, 11]. In the present study, PGS detected EIBS in vivo prior to tumor formation. The ‘Early tumor formers’ a subset of AOM-treated mice in the AOM alone group had lesions on colonoscopy by wk. 20. However, these mice exhibited blood supply changes as early as week 12, including a marked increase in rectal [Hb] (Fig. 4a), decrease in BVR (Fig. 5a) and aberrations in microvascular structure observed with CLE imaging (Fig. 6). In the CLE images, small, ectactic vessels resemble the neovascular sprouting we demonstrated in previous studies of tumor angiogenesis [31]. Taken together, these results indicate that neoangiogenesis in the mucosal microcirculation precedes tumor formation, demonstrating some of the earliest blood vessel changes observed prior to tumor development.

The neoplastic angiogenic switch is generally regarded as occurring when a tumor outstrips the native blood supply and thereby induces new vessel networks in response to hypoxia [15]. However, the data presented here show that vascular abnormalities mimicking neoangiogenesis precede adenoma development. This indicates the angiogenic switch might actually be triggered in the earliest stages of tumor development, rather than later when the tumor exceeds its current blood supply. VEGF is an important growth factor implicated in angiogenesis and was shown to be up-regulated in the colonic mucosa as a result of the RAS-modulation and AOM-treatment [21]. While additional studies are needed to fully understand the molecular mechanisms driving early neovascularization, these changes are consistent with the concept of field effects preceding malignant transformation. Animals treated with chemical carcinogen are predicted to develop adenomas and, in that sense, they are “at risk” for neoplasia. The microvascular changes shown in Fig. 6 are widespread throughout the normal appearing mucosa of AOM-treated mice and arise prior to neoplastic lesions. Furthermore, rectal [Hb] is increased regardless of the location of focal malignant lesions. These widespread vascular changes that precede tumor development are markers of field effects. Further understanding of pathways driving early vascular changes preceding tumor emergence could lead to improved diagnostic approaches and anti-angiogenic strategies to detect or prevent colonic neoplasia.

The role of AngII in angiogenesis has been studied extensively and recent studies suggest a clinical potential of the RAS as a druggable target for a wide range of cancers [16, 23, 35]. However, only recently, the RAS link to colon cancer has been investigated. In a murine model of CRC liver metastasis, Neo et al. reported a significant decrease in the number of tumors and tumor volume with treatment using either an ARB or ACE inhibitor [36]. In a subsequent study with the same cancer model, these investigators demonstrated a distinct cancer cell-associated RAS expression with increased expression of AT1R, and again, ACE inhibitor treatment led to a reduced tumor volume and decrease in AT1R expression [37]. In another murine model using AOM to induce colon carcinogenesis, Kubota et al. demonstrated that administration of either ARBs or ACE inhibitors significantly reduced development of aberrant crypt foci (ACF) preneoplastic lesions in the AOM model [38]. In a retrospective study focused on colon cancer and long-term use of ACE inhibitors, Kedika et al. found a reduction in the recurrence and development of new advanced adenomatous polyps in patients who received a follow-up colonoscopy for a previously diagnosed adenomatous polyp and were continuously receiving lisinopril, an ACE inhibitor [39]. The present study shows that the RAS inhibitor, Losartan, suppressed tumor multiplicity and down-regulated VEGF protein levels. Additionally, the AOM-treated mice receiving Losartan did not exhibit the same increase in rectal [Hb] for ‘Late tumor formers’ nor the extreme microvascular changes in varying vessel size and structure as the AOM alone group, pointing to the potential of ARBs to mitigate these effects. These findings provide further support to investigate the anti-angiogenesis potential of the RAS inhibition in colonic carcinogenesis.

In addition to the critical time-point these studies uncover the potential importance of a critical blood supply “threshold” that is required to support the progression of pre-dysplastic mucosa into tumors. Our data show that AOM-treated mice with RAS manipulation did not exhibit the same EIBS trends associated with early tumors. This observation appears to support the hypothesis of the existence of a critical threshold. Since angiogenesis is required for unrestricted tumor growth, individuals that have pre-existing elevated blood supply or overexpression of the key regulators (i.e. AngII or VEGF) may already have sufficient levels of blood supply to foster tumor promotion without the pre-dysplastic mucosa having to trigger an early angiogenic switch. In this case, the tissue may not need to acquire an early increase in blood supply associated with neoplasia. Conversely, if blood supply levels were depressed below the threshold, angiogenic factors might need to be induced in order for the tumor to grow and this might manifest as EIBS. This hypothesis might explain a previously reported observation that colon neoplasia is more frequently associated with EIBS in subjects and colon segments which tend to have lower baseline levels of blood supply such as proximal neoplasia in females. This hypothesis will also be important for understanding EIBS in individuals who have preexisting gastrointestinal conditions which alter their baseline blood supply levels.

The AOM + AngII group did not show the same increase with the rectal [Hb] as the AOM alone group, yet had 100% (8/8) tumor incidence within the group. It is possible that the vasoconstrictive properties of AngII coupled with the expected increase in angiogenesis from AOM carcinogen masked changes in rectal [Hb]. Similarly, the complex interplay between the anti-angiogenic effects of Losartan and tumor driven angiogenesis may affect field carcinogenesis in a completely different and still unexplained manner than described for the standard AOM model used in previous studies. Rectal PGS measurements may not be able to detect these subtleties. Further studies will be needed to understand if additional biomarkers can enhance PGS detection. In part, these results stimulated the analysis of the change in [Hb] with respect to absolute values from week 2 (shown in Fig. 7). The mice demonstrating lower baseline [Hb] at the time of AOM treatment showed a rectal [Hb], that continued to increase over time as tumors developed. In contrast, mice with [Hb] close to baseline controls track with the RAS-unmodulated baseline group throughout the study. Perhaps the latter mice have sufficient blood supply for tumor growth, which would suggest that the [Hb] levels of RAS-unmodulated saline controls are sufficient to promote tumorigenesis. Characterizing the factors that determine an individual critical threshold of blood supply that accurately predicts the risk for developing a lesion could have clinical relevance for identifying these “high-risk” patients.

There are several limitations in this study. For this study we used a single model, the A/J strain specifically for its susceptibility to the AOM carcinogen and expected tumor incidence. While the AOM carcinogen model is a widely used model for colorectal cancer, it would be ideal to evaluate the role of blood supply in other murine and rodent strains or tumor models including the conditional Apc+/Min mouse model [5]. In this longitudinal study, we measured only PGS and CLE at several time-points. Correlating the observed microvascular changes with other critical tissues changes (eg. dysplastic ACF development) could shed more light on early angiogenic events as an indicator of tumor development. We limited our study to VEGF-A, but other members of the VEGF family may also be involved. There are also other angiogenic pathways beyond VEGF that need to be explored including PDGF and matrix metalloproteases. Furthermore, a detailed time course of VEGF-A expression will be useful to better define the potential causal relationship between VEGF-A and microvascular abnormalities. Despite these limitations, our initial promising results highlight the importance of the renin-angiotensin system during colonic tumorigenesis and uncover new aspects of the role of the RAS on microvessel architecture in normal and transforming tissues.

## Conclusions

In conclusion, this study further supports the concept of an early increase in rectal blood supply in association with the risk of developing colonic tumors. We demonstrated angiogenic alterations prior to tumor formation including changes in VEGF-A signaling, microvascular architecture, and microvascular blood supply. We also demonstrated that Losartan mitigated these angiogenic alterations, and lowered tumor multiplicity. This work should stimulate further investigations into the molecular mechanisms that regulate neovascularization and drive tumor angiogenesis, which could ultimately determine the risk of tumor development and the prognosis of tumor progression.

## Abbreviations

[Hb]: Hemoglobin concentration
ACE: Angiotensin-converting-enzyme
AngII: Angiotensin-II
ANOVA: Analysis of variance
AOM: Azoxymethane
ARB: Angiotensin receptor blocker
AT1R: AT1 subtype receptor
BVR: Blood vessel radius
CLE: Confocal laser endomicroscopy
CRC: Colorectal cancer
DIL: 1,1′-dioctadecyl-3,3,3′,3′-tetramethylindocarbocyanine perchlorate
EIBS: Early increase in blood supply
IACUC: Institutional Animal Care and Use Committee
IHC: Immunohistochemistry
MIN: Multiple intestinal neoplasia
MVD: Microvascular density
NOS: Nitric oxide synthase
PGS: Polarization gated spectroscopy
RAS: Renin angiotensin system
TBST: Tris-buffered saline with 0.1% Tween-20
VEGF: Vascular endothelial growth factor
